# Curcumin in Autoimmune and Rheumatic Diseases

**DOI:** 10.3390/nu11051004

**Published:** 2019-05-02

**Authors:** Melissa Yang, Umair Akbar, Chandra Mohan

**Affiliations:** Department of Biomedical Engineering, University of Houston, 3517 Cullen Blvd, Room 2004, Houston, TX 77204, USA; Melissa.Y.Yang@uth.tmc.edu (M.Y.); Umair@jama.org.uk (U.A.)

**Keywords:** osteoarthritis, diabetes, ulcerative colitis, lupus, rheumatic

## Abstract

Over recent decades, many clinical trials on curcumin supplementation have been conducted on various autoimmune diseases including osteoarthritis, type 2 diabetes, and ulcerative colitis patients. This review attempts to summarize the highlights from these clinical trials. The efficacy of curcumin either alone or in conjunction with existing treatment was evaluated. Sixteen clinical trials have been conducted in osteoarthritis, 14 of which yielded significant improvements in multiple disease parameters. Eight trials have been conducted in type 2 diabetes, all yielding significant improvement in clinical or laboratory outcomes. Three trials were in ulcerative colitis, two of which yielded significant improvement in at least one clinical outcome. Additionally, two clinical trials on rheumatoid arthritis, one clinical trial on lupus nephritis, and two clinical trials on multiple sclerosis resulted in inconclusive results. Longer duration, larger cohort size, and multiple dosage arm trials are warranted to establish the long term benefits of curcumin supplementation. Multiple mechanisms of action of curcumin on these diseases have been researched, including the modulation of the eicosanoid pathway towards a more anti-inflammatory pathway, and the modulation of serum lipid levels towards a favorable profile. Overall, curcumin supplementation emerges as an effective therapeutic agent with minimal-to-no side effects, which can be added in conjunction to current standard of care.

## 1. Introduction

Curcumin is the main component of turmeric, also known as the *Curcuma longa*, which belongs to the ginger family, Zingiberaceae [1]. Curcumin is commonly used in Indian and Asian cooking as a spice for its flavor and yellow color profile. In addition to its consumption due to flavor, curcumin has been used for its medicinal properties for thousands of years. Most commonly, curcumin was used for wound healing in India [2].

The curcuminoids found in turmeric are curcumin, desmethoxycurcumin, and bisdemethoxycurcumin, with curcumin being the main active component. Curcumin was first isolated from turmeric in 1815 [3]. Curcumin gives turmeric its yellow color and is known to possess most of the therapeutic effects of turmeric. In 1937, the first article published on the use of curcumin in treating human disease cited its beneficial effects in biliary disease [4]. Since then, continued research has shown that curcumin can alleviate a number of human diseases.

Studies performed on animals have shown a direct relationship between increased cellular curcumin concentrations and its ability to modulate inflammatory mediators [5]. Experimental studies on cell lines and humans have confirmed the findings from animal studies, demonstrating that curcumin plays a role in anti-inflammatory response via inhibition of the COX-2 pathway and NF-kB activation [6]. In more recent years, numerous studies have shown that curcumin possesses potential anti-inflammatory, anti-oxidant, anti-diabetic, and anti-cancer properties [3]. These studies indicate that curcumin acts on numerous targets with various mechanisms of action, altering enzyme, receptor, and transcription factor activity [7]. In addition, curcumin administration has reported nearly no side effects, making it a potential alternative to NSAIDs and other medications with known severe adverse effects [8]. Previous systemic reviews on curcumin in rheumatic diseases have either focused on one specific disease or on fewer studies than this review. Examining all studies from different rheumatic diseases will allow a better opportunity to evaluate how curcumin elicits its therapeutic potential via various mechanisms of action in a multitude of disease processes. This article reviews studies on the efficacy of curcumin in osteoarthritis (OA), type 2 diabetes mellitus (T2DM), ulcerative colitis (UC), rheumatoid arthritis (RA), lupus nephritis (LN), and multiple sclerosis (MS).

## 2. Materials and Methods

A literature search on PubMed was conducted to gather articles in this study using the MeSH terms (curcumin or curcuminoid or *curcuma*) AND (osteoarthritis or type 2 diabetes or ulcerative colitis or lupus nephritis or rheumatoid arthritis or multiple sclerosis). The search identified 473 articles, of which 139 were on OA, 135 were on T2DM, 95 were on UC, 88 were on RA, 6 were on LN, and 32 were on MS.

From this initial search, the following criteria were used to determine article inclusion in this review: (1) original research articles written in English; (2) randomized trial; (3) included control group consisting of at least a placebo or multiple curcumin dosage arms; and (4) quantifiable daily oral dosage of curcumin. Only full texts of relevant articles were included after screening of titles and abstracts. From this process a total of 32 articles were included in this review.

## 3. Results

### 3.1. Impact of Curcumin Supplementation in Osteoarthritis

A total of 16 published articles on the efficacy of curcumin supplementation in OA met the inclusion criteria for this review [9,10,11,12,13,14,15,16,17,18,19,20,21,22,23], as summarized in Table 1. All studies were randomized, placebo controlled trials. Trial length ranged from 6 to 40 weeks. The amount of curcumin ingested ranged from 100–2000 mg daily. Among the clinically effective studies—which include studies reporting either significant improvement in clinical or laboratory outcomes—the mean curcumin dosage was 834.29 mg, with an interquartile range of 1300 mg. Most commonly, curcumin used in the trials are sourced from curcuminoids isolated from the rhizome of turmeric. One study specified the use of pure curcuminoid [13], while seven studies specified the formulation of curcuminoids used, consisting of a ratio of curcumin:desmethoxycurcumin:bisdemethoxycurcumin [9,10,11,12,16,19,23]. In addition, two studies administered oral *Boswellia serrata* in conjunction with curcumin supplementation [19,24]. *Boswellia serrata* is a gum resin extract which possesses anti-inflammatory and anti-arthritic effects by decreasing glycosaminoglycan levels, which are necessary for cartilage repair [24]. Clinical outcomes measured in the majority of studies included The Western Ontario and McMaster Universities Arthritis Index (WOMAC), walking distance, visual analogue scale (VAS), Karnofsky performance scale, Lequesne’s pain functional index (LPFI), Clinical Global Impression (CGI), and Knee injury and Osteoarthritis Outcome Score (KOOS).

All but two studies measured clinical outcomes [11,13]. These two studies solely measured inflammatory markers and oxidative stress indicators. In 13 of the studies, dietary curcumin intake resulted in improvement of at least 2 clinical measures [9,10,12,14,15,16,17,18,19,21,22,23,24]. Furthermore, seven studies showed improvement of at least three clinical measures [9,14,16,17,18,19,22]. Studies with positive clinical outcomes most commonly reported increased walking distance and decreased WOMAC scores. One study reported no significant improvements of pain and function, although there was a tendency towards improved scores [20]. A total of eight studies reported laboratory findings after curcumin supplementation [10,11,12,13,19,21,22,24]. Five of the eight studies reported either significantly decreased inflammatory markers or oxidative stress markers [10,11,21,22,24]. In three studies, patients continued NSAID use in conjunction to the administered curcumin supplementation [10,20,22].

### 3.2. Examination of Individual Studies

#### 3.2.1. NSAID comparison

Four studies compared the effectiveness of NSAID use versus curcumin supplementation in OA [15,16,19,23]. Nonsteroidal anti-inflammatory drugs exert their action by inhibiting prostaglandin synthesizing enzymes. In particular, the COX-1 and COX-2 pathways are inhibited, which can result in adverse effects such as GI pain. In addition, NSAIDs are documented to inhibit the lipoxygenase pathway and interfere with G protein signal transduction [25]. In 2014, Kuptniratsaikul et al. compared the effectiveness of curcumin with ibuprofen [16]. A total of 367 patients with knee osteoarthritis were split into two groups: one received 1200 mg daily ibuprofen while the other received 1500 mg daily of curcumin for a duration of 4 weeks. Both treatment groups exhibited significant improvement in clinical parameters of WOMAC pain and function scores. Additionally, the number of patients with adverse events were the same in both groups. However, there was a significantly higher number of patients with abdominal pain in the ibuprofen group. Findings from this study indicate that use of curcumin supplementation for OA is as effective as ibuprofen (NSAIDs) with fever gastrointestinal adverse effects. This is supported by results from a 2014 study by Nakagawa et al., which reported a decrease in use of NSAIDs and other painkillers in the curcumin treatment group as well as decreased GI complaints [15].

Kuptniratsaikul et al. conducted a similar study in 2009 in which patients received either 800 mg daily ibuprofen or 2 g daily curcumin extract for 6 weeks [23]. There was no difference in improvement in main outcomes such as pain while walking and increased knee function, except for pain on stairs. Additionally, no significant difference in adverse effects between both groups was reported.

In 2013, Kizhakkedath et al. tested the effectiveness of curcumin and boswellia combination supplement versus celecoxib, a selective COX-2 pathway inhibitor [19]. The 500 mg curcumin and boswellia formulation contained 350 mg curcumin extract and 150 mg *Boswellia serrata* extract. Patients were split into two groups, taking either 1000 mg curcumin-boswellia mixture daily or 200 mg celecoxib daily for 12 weeks. Both treatment groups experienced significant improvement in pain scores, walking distance, joint line tenderness, and crepitus but there was no significant difference between the two groups. However, the curcumin and boswellia formulation showed greater improvement in pain scores, walking distance, and joint line tenderness in comparison to celecoxib. Treatment was well tolerated without toxicities or adverse effects. Overall, these studies support the comparable efficacy of curcumin in the treatment of OA compared to NSAIDs, with decreased adverse effects.

#### 3.2.2. Oxidative Stress and Inflammatory Markers

A total of five studies measured inflammatory markers after curcumin supplementation in OA [10,12,13,21,22]. Of these studies, three reported a decrease in inflammatory markers and two reported no change in inflammatory markers. Sterzi et al. in 2016 was one study that did not report changes in inflammatory markers [12]. Patients were administered a combination of glucosamine hydrochloride, chondroitin sulfate and bio-curcumin, which consisted of 100 mg curcumin daily for 12 weeks. Inflammatory markers CRP and ESR did not change in the treatment group. However, one study suggests that glycosaminoglycans have only a short term effect on pain relief compared to the long treatment duration, which may explain why there was no improvement in inflammatory markers or knee ROM [26]. In addition, the 100 mg curcumin dosage is comparably lower than other trials with positive outcomes included in this review. However, in 2015, Rahimnia et al. conducted a study in which patients were administered 1500 mg curcumin daily for 40 weeks and did not report significant improvement in inflammatory markers as well [13].

In 2016, Srivastava et al. conducted a 16-week study where patients consumed 500 mg curcumin daily [10]. Inflammatory IL- 1ß values significantly decreased in the curcumin group. Similarly, in 2010 Belcaro et al. also reported a decrease in IL-1ß in the treatment group [22]. Patients in this study were administered 1000 mg Meriva, a curcumin-phosphatidylcholine complex with improved stability and oral absorption, which contains a dosage of 200 mg daily of curcumin for 32 weeks. The treatment group used Meriva with the “best available treatment” as defined by the patient’s general practitioner. In addition to decreased IL- 1ß levels, all tested inflammatory markers decreased, which include ESR, IL-6, sCD40L, and sVCAM-1. Belcaro et al. conducted another study in 2010 with the same curcumin dosing on 50 patients for 12 weeks, and reported a decrease in CRP, an inflammatory marker [21]. This shows that curcumin remarkably decreases inflammatory players involved in OA.

Three studies reported decreased oxidative stress markers in OA patients treated with curcumin. Srivastava et al. reported decreased GSH, ROS, and MDA [10]. These results are supported by a study by Panahi et al. in 2016 which also reported decreased MDA levels as well as increased GSH and SOD [11]. GSH and SOD are antioxidants that increased after a 6-week treatment of 1500 mg daily curcumin. In addition, Badria et al. also reported a decrease in oxidative stress markers after 12 weeks of treatment with 500 mg daily curcumin [24].

### 3.3. Impact of Curcumin Supplementation on Type 2 Diabetes

There have been at least 8 studies conducted on the efficacy of curcumin supplements on type 2 diabetes mellitus [27,28,29,30,31,32,33,34], as summarized in Table 2. Included studies were placebo controlled and randomized clinical trials. The average duration of curcumin intake ranged from 4 to 36 weeks. The amount of curcumin intake ranged from 200 to 1500 mg daily. In the studies where curcumin supplementation proved to be clinically effective, the mean dosage was 570.79 mg and the interquartile range was 700 mg. Six studies specified use of varying ratios of natural curcuminoid mixtures of curcuminoid:desmethoxycurcuminoid:bisdemethoxycurcumin, while the remaining study utilized encapsulated turmeric rhizomes. Six of the seven studies utilized patients already diagnosed with T2DM, while one study used a pre-diabetic patient population [30]. The laboratory outcomes measured in most studies included: blood glucose, TNF-, IL-6, and HOMA-IR.

A total of five studies reported significantly improved clinical outcomes in the curcumin group [27,30,31,32,34]. Six studies reported three or more improved laboratory measures [27,28,29,30,33,34]. Three included studies assessed the effect of curcumin supplementation on sequelae associated with T2DM such as atherosclerosis [27] and diabetic microangiopathy [31,32]. Overall, the studies showed curcumin supplementation possesses anti-diabetic effects and improves T2DM parameters in patients.

#### 3.3.1. Examination of Individual Studies

Two of the seven studies report anti-diabetic outcomes of glucose lowering and improved ß cell function [29,30]. In 2012, Cheungsamarn et al. conducted a 36-week study with 240 pre-diabetic patients [30]. Patients were split into two groups: the curcumin group who consumed 1500 mg curcumin daily, while the placebo group consumed placebo capsules. At the end of the study, 16.4% of subjects in the placebo group were diagnosed with T2DM and no patients in the curcumin group were diagnosed. Curcumin improved ß cell function, indicated by a significant increase in HOMA- ß and significant decreases in C-peptide and HOMA-IR. Curcumin also reduced inflammation by significantly increasing adiponectin, an anti-inflammatory cytokine. Interestingly, there was a slight reduction in mean body weight and WC in the curcumin group by the last follow up visit. No serious adverse effects were noted, however a few noted minor symptoms of itching, constipation, or vertigo.

In 2013, Na LX et al. conducted a study with 100 overweight/obese type 2 diabetes patients for 12 months [29]. Curcumin supplementation was administered as 300 mg daily. By the end of treatment, patients in the curcumin group had significantly lower fasting blood glucose levels, HbA1c, HOMA-IR, FFA, and triglycerides and a significant increase in LPL activity, which promotes fatty acid oxidation and utilization. This suggests a glucose lowering effect of curcumin due to decreased serum FFA.

In 2014, Na LX et al. investigated the role of serum adipocyte fatty acid binding protein (A-FABP) in regulating fatty acid metabolism and metabolic syndrome in patients taking curcumin [28]. A-FABP is a cytoplasmic protein implicated in intracellular fatty acid trafficking. Animal studies have shown a deficiency in A-FABP can improve glucose and lipid metabolism in obese and ApoE deficient mice [35]. Curcumin supplementation of 300 mg daily for 12 weeks led to significant decreases in inflammation and oxidative stress parameters of CRP, TNF-ɑ, and IL-6. Additionally, significant decreases in serum A-FABP levels positively correlated with changes in serum FFA levels. Thus, serum A-FABP reduction by curcumin is associated with improved metabolic parameters in T2DM.

#### 3.3.2. Oxidative Stress and T2DM

Some studies assessed the effect of curcumin supplementation on suppressing inflammatory cytokines and oxidative stress in T2DM patients [28,33,34]. In two of the studies, there was a significant decrease in TNF-ɑ and IL-6 [28,34]. Usharani et al. compared T2DM patients treated with either placebo, curcumin supplements, or cholesterol lowering drug atorvastatin [34]. The curcumin group was administered 300 mg daily and the atorvastatin group was administered at 10 mg daily. Post treatment, patients receiving curcumin or atorvastatin showed significant improvement in endothelial function and significant decreases in levels of malondialdhyde, ET-1, IL-6, and TNF-ɑ. Curcumin had a favorable effect similar to atorvastatin in reducing inflammatory cytokines and oxidative stress markers. 

#### 3.3.3. T2DM Associated Sequelae

Four studies evaluated complications associated with T2DM [27,31,32,33]. In two studies, curcumin supplementation was used to treat diabetic microangiopathy. In studies by Steigerwalt et al. and Appendino et. al, curcumin was administered in the form of Meriva, a lecithinized curcumin delivery system, of 200 mg and 400 mg curcumin per day, respectively [31,32]. Both studies reported a significant improvement in microangiopathy and venoarteriolar response and a decrease in edema score. The diabetes nephropathy study was conducted with 40 patients with a dosage of 66.3 mg daily curcumin for 12 weeks [33]. Laboratory outcomes showed a decrease in inflammatory markers TGF-ß, IL-8, and proteinuria. In 2014, Cheungsarman et al. conducted a study to investigate the effect of curcumin on atherosclerosis in T2DM patients [27]. Outcomes showed an improvement in inflammatory and metabolic profiles as well as a significant decrease in pulse wave velocity, a parameter for atherosclerosis. Not only does curcumin supplementation decrease incidence of general T2DM parameters, these studies demonstrate curcumin’s effect on improving and limiting diabetes associated complications.

### 3.4. Impact of Curcumin Supplementation on Ulcerative Colitis

At least three studies have been conducted to assess the role of curcumin supplementation in UC [36,37,38], as summarized in Table 3. All studies were double blind, randomized, and placebo controlled. The duration of studies ranged from 4 weeks to 24 weeks. The amount of curcumin ingested ranged from 140 mg to 3000 daily. The mean dosage of curcumin for the clinically effective studies was 2500 mg. All three studies specified the use of purified curcuminoid as the source of supplementation. Clinical outcomes include clinical remission, clinical activity index, and endoscopy and relapse frequency. In all studies, oral mesalamine or sulfasalazine medication was continued.

Of the three studies, two reported a decrease in clinical activity or an increase in clinical remission while one study reported curcumin as ineffective in reducing remission. The clinical outcomes measured include clinical remission and endoscopy index. In two studies, dietary curcumin supplementation resulted in improvements of at least three clinical measurements [37,38].

#### 3.4.1. Examination of Individual Studies

Two of the three studies reported significant improvement in disease remission [37,38]. In 2006, Hanai et al. reported findings that oral curcumin maintained remission and decreased relapse and clinical activity in patients with quiescent UC [38]. Results include improvement in CAI and EI scores. In 2015, Lang et al. reported that oral curcumin was effective in inducing remission in patients with active mild to moderate UC [37]. Patients were administered curcumin at 3000 mg daily for one month, resulting in significant improvement in rate of clinical remission by week 4, clinical response, and endoscopic remission.

In 2017, Kedia et al. reported that oral curcumin was not effective in inducing remission in mild to moderate UC [36]. Patients received 450 mg daily of oral curcumin for 8 weeks. In comparison to placebo, there was no significant difference in rates of clinical remission, clinical response, mucosal healing, or treatment failure. In comparison, patients in Kedia’s study received a low dose of 450 mg daily while patients in Lang and Hanai’s trials received 3000 and 2000 mg daily, respectively. These results show that low dose oral curcumin supplementation is ineffective in inducing remission in mild to moderate UC while higher doses of curcumin show promising effects.

### 3.5. Impact of Curcumin Supplementation on Other Diseases

A total of three studies in other rheumatic diseases met the criteria our study, which included two studies on rheumatoid arthritis and one on lupus nephritis [39,40,41], as summarized in Table 4. Of the two RA studies, one study of 8-week duration showed a decrease in clinical outcomes in the curcumin group, with decreased ACR, DAS, and VAS scores [39,40]. There was a significant decrease in CRP inflammatory marker, but no significant improvement in ESR or other blood parameters. In the other study, there was no improvement in DAS score, which may be attributed to the year the study was conducted (1980) and the short 2-week trial duration. The LN study showed significant improvement in laboratory outcomes, in which patients were administered 66.3 mg curcumin daily for 12 weeks [41]. Outcomes reported include decrease proteinuria, systolic blood pressure, and hematuria in the curcumin group. Due to the limited number of studies conducted on RA and LN, each with limited numbers of subjects, results are inconclusive. More well-designed trials with sufficient dosage of curcumin and sufficient cohort sizes are necessary to fully evaluate and understand the effects of curcumin supplementation on these diseases.

Two clinical trials on multiple sclerosis assessed laboratory outcomes of curcumin supplementation. Oral nanocurcumin was administered at 80 mg daily for 26 weeks in both studies. Results from the first study showed that nanocurcumin is able to restore the expression pattern of dysregulated mRNA [42]. In the other study, there was a significant decrease in Th17 cell parameters and IL-17 mRNA expression, which may improve disease progression in MS patients [43]. A literature search yielded no trials which assessed clinical outcomes in multiple sclerosis, which warrants the need for future clinical studies. Another search was performed on curcumin supplementation in additional autoimmune diseases, but this search did not produce any hits.

### 3.6. Curcumin Side Effects 

Overall, oral curcumin supplementation is very well tolerated in OA, T2DM, and UC patients with only minor side effects documented. In total, seven studies reported adverse effects in OA patients, two studies in T2DM patients, and two studies in UC patients. Of the clinical trials that reported adverse effects, the following side effects occurred in decreasing frequency: mild gastrointestinal symptoms (such as dyspepsia, meteorism, bloating, and gastroesophageal reflux), nausea and vomiting, edema, loose stool, constipation, increase in stool frequency, and hot flashes. In all studies, the frequency of adverse events was evenly distributed and not significantly different between the placebo group and treatment groups. It is noteworthy that in one ulcerative colitis study, serious adverse events were observed in both the active treatment arm and placebo group, which led to early withdrawal from the study [37]. One patient taking curcumin supplements was hospitalized with abdominal pain due to a peptic ulcer. However, the rate of severe adverse events was not different between the two groups. This indicates that the reported minor or major effects may not be caused by curcumin supplementation; rather, other disease mechanisms may have contributed to these events.

Several studies compared adverse events in curcumin groups versus NSAID groups. In Kuptniratsaikul et al., there was no significant difference in the number of events between the two groups, however the ibuprofen group had increase frequency of all adverse events compared to the curcumin group except for loose stool. On the other hand, another study reported a significant decrease in GI complaints in patients taking Meriva plus “the best available treatment” for OA compared to controls, who used best available treatment only. While adverse effects are extremity minimal in curcumin supplementation, the results from small sample size trials cannot be used to make a clear conclusion on severity and frequency of side effects. Investigation of curcumin side effects would benefit from larger, more systematic patient studies.

### 3.7. Overview of Mechanisms of Action

Curcumin modulates inflammation through many cellular targets, although the exact mechanisms are unclear. Curcumin must first undergo oxidative activation to exert its oxidative and anti-inflammatory abilities [44]. Through a series of conjugations, reductions, and metabolic pathways, various curcumin metabolites are produced. The majority of studies indicate that curcumin exert its effects by binding to proteins COX-2, lipoxygenase, and GSK3b. One main pathway curcumin affects is arachidonic acid metabolism, leading to decreased COX-2 expression and subsequent prostaglandin and thromboxane synthesis, and inhibition of 5-LOX activity and subsequent leukotriene synthesis [45]. Decreased expression of these compounds attributes to the anti-inflammatory effects of curcumin. The mechanism of action of curcumin is similar to the mechanism of NSAIDs, a common treatment for osteoarthritis. NSAIDs such as celecoxib inhibit the inducible COX-2 isoenzyme, which modulates inflammation. However, the cardiovascular safety of COX-2 inhibitors and non-specific attributes of NSAIDs have become more concerning as trials suggest that COX-2 inhibitors reduce prostacyclin production, an antithrombotic product [46]. Thus, the use of curcumin in inflammatory diseases is a plausible alternative due to decreased side effects. Other studies report that curcumin decreases inflammation in osteoarthritis synovial cells by inhibiting matrix metalloproteinase-3 (MMP3) expression [47]. MMP3 breaks down extracellular matrix proteins in normal physiological processes as well as disease processes such as arthritis and tumor proliferation. By inhibiting MMP3 in osteoarthritis, inflammation and cartilage degradation should decrease.

Curcumin also shows direct effects in T2DM progression due to its anti-inflammatory effects. Tetrahydrocurcumin, an active metabolite of curcumin reduces HMG CoA reductase activity, lowering serum and liver cholesterol, triglycerides, free fatty acids, VLDL, and LDL [48]. Curcumin attenuates NF-κB activation and macrophage accumulation in adipose disuse, decreasing insulin resistance, and hyperglycemia development [49]. 

Additionally, curcumin can modulate intracellular redox states, influencing a number of cellular processes by affecting transcription factor activation. Specific transcription factors affected by curcumin include NF-AT, AP-1, STAT, p53, kinases, and cytokine release [50]. Thus, curcumin influences both the innate and adaptive immune response and can modulate immune cells such as B cells, T cells, and macrophages.

#### 3.7.1. Additional Benefits of Curcumin

The therapeutic effects of curcumin extend beyond the scope of rheumatic diseases. Numerous research studies have investigated the anti-cancer properties of curcumin. Curcumin has the ability to suppress tumor cell proliferation and downregulate transcription factors Nf-κB, AP-1, and Egr-1 [51]. In addition, curcumin exerts effects on the cell cycle to inhibit growth factor induced cell proliferation via IL-2, PDGF, and PHA [52]. Curcumin possesses in vivo apoptotic properties in cancer cell lines such as leukemia, breast cancer, and prostate cancer [53,54,55].

Additionally, research has been conducted on the neuroprotective effects of curcumin. Curcumin can inhibit histone acetyltransferase and limit amyloid-forming peptide aggregation common in neurodegenerative diseases such as Alzheimer’s [56,57]. Furthermore, a meta-analysis found that curcumin supplementation for depressed patients ameliorated depressive symptoms [58].

## 4. Discussion

Curcumin has been used in Indian and Asian medicine for its therapeutic abilities for thousands of years. Recent studies provide evidence that increased curcumin levels can modify cellular disease mechanisms toward a more anti-inflammatory profile. This mainly occurs by inhibition of the COX and lipoxygenase pathway. Additionally, curcumin suppresses NF-κB pathway to decrease inflammation. In addition to its anti-inflammatory effects, curcumin also exerts anti-oxidative capacity. The involvement of these pathways in normal rheumatic disease progression could possibly explain curcumin’s therapeutic effects targeting these disease processes.

The curcuminoids found in turmeric are curcumin, desmethoxycurcumin, and bisdemethoxycurcumin, with curcumin being the main active component. Studies in this review used dietary curcumin supplements that varied in ratio of these components, however all capsules had more abundant curcumin compared to the other two molecules. Another active component of turmeric is turmeric essential oils, composed mainly of sesquiterpenes. Curcuma oil was incorporated into curcumin capsules in two OA studies and may have added to the clinical efficacy of curcuminoids [9,19]. Although curcumin supplementation has shown therapeutic efficacy in many clinical trials, it possesses limited bioavailability due to poor absorption, and rapid metabolism and elimination which limits its effects [7]. To increase oral curcumin bioavailability, many studies administered either an altered form of curcumin or administered compounds in conjunction with curcumin. Three studies added piperine to the curcumin treatment regimen [11,14,15]. Piperine is a major component of black pepper shown to increase curcumin bioavailability by 2000% [59]. Alternatively, other studies administered a modified form of curcumin with increased bioavailability. Meriva was administered in three OA studies and two T2DM studies and Theracurmin was administered in 1 OA study [15,17,21,22,31,32]. While these administration methods increase bioavailability of curcumin, it should be noted that clinically efficacious results were not limited to these trials, as studies using standard curcuminoid capsules also resulted in significantly improved outcomes in treatment groups. Furthermore, the variable preparation of curcumin from different sources across studies makes it difficult to draw comparisons of dosage and results between all studies for a disease, potentially flawing the interquartile range and mean dosage calculations reported here.

Osteoarthritis has been the most researched rheumatic disease of the those included in this review. Many positive outcome clinical trials in OA present solid evidence of curcumin’s beneficial role in disease progression. In addition, curcumin shows similar efficacy to common NSAIDs such as ibuprofen, which cause serious GI side effects. Conversely, curcumin has shown to have little or no side effects. While there are several short term positive outcome studies conducted on T2DM patients, longer term study trials with larger patient populations are necessary to strengthen the association of curcumin supplementation and diabetes. In addition, follow up evaluations should be conducted in future studies to evaluate long lasting effects. Results are mixed for ulcerative colitis studies, as the dosages may not have been equally high in all studies. More long term studies with higher daily doses are clearly warranted in this disease. Finally, few trials have been published on other rheumatic diseases. Additional research is warranted in rheumatoid arthritis, lupus, and other autoimmune diseases. In terms of curcumin supplementation, another area of research is increasing the bioavailability of curcumin. Some studies administered specially formulated curcumin with increased bioavailability, but more research is warranted. Other ingredients in turmeric also merit detailed evaluation. Lastly, the mechanisms of action of curcumin warrant deeper investigation, using currently available OMICs technologies.

## Figures and Tables

**Table 1:** Clinical efficacy of curcumin supplementation in osteoarthritis

Study	Study Design	No. of Patients	Study Duration (weeks)	Medications	Daily Curcuminoid Intake (mg/day)	Clinical Outcomes in Curcumin Group	Laboratory Outcomes in Curcumin Group
2018 Haroyan [9]	DB, PC, RCT	66	12		1500	↑ Walk speed ↓WOMAC, TUG time	N/A
2016 Srivastava [10]	DB, PC, RCT	160	16	NSAID continued	500	↓ VAS, WOMAC	↓ IL-1B, ROS, MDA
2016 Panahi [11]	DB, PC, RCT	40	6		1500	N/A	↑ GSH, SOD↓ MDA
2016 Sterzi S [12]	DB, PC, RCT	53	12		100	⇄ Knee ROM. ↓ LPFI, VAS	⇄ Inflammatory markers
2015 Rahimnia [13]	DB, PC, RCT	40	40		1500	N/A	⇄ Inflammatory markers
2014 Panahi [14]	DB, PC, RCT	40	6		1500	↓ LPFI, VAS, WOMAC	N/A
2014 Nakagawa [15]	DB, PC, RCT	50	8	NSAID continued	180	↓ Celecoxib use, VAS	N/A
2014 Kuptniratsaikul [16]	DB, RCT	367	4	NSAID continued	1500	↑ PhyGA. ↓ GI AE, WOMAC	N/A
2014 Belcaro [17]	OBS, PC	124	16		500	↑ Walking distance. ↓ Karnofsky, WOMAC, general pain	N/A
2013 Madhu [18]	SB, PC, RCT	120	6		1000	↓VAS, WOMAC, CGIC	N/A
2013 Kizhakkedath [19]	PC, RCT	30	12	NSAID continued	500	↑ Walk distance.↓ Joint tenderness, Crepitus, Knee ROM	⇄ Hemogram measurement, RFT
2012 Pinsornsak [20]	DB, PC, RCT,	88	12	NSAID continued as needed	1000	⇄ KOOS, VAS	N/A
2010 Belcaro [21]	DB, PC, RCT	50	12		200	↑ Walk distance. ↓ WOMAC	↓ CRP
2010 Belcaro [22]	DB, PC, RCT	100	32	NSAID continued as needed	200	↑ Walk distance, mobility, Karnofsky. ↓ NSAID use, WOMAC	↓ ESR, IL-1B, IL-6, sCD40L, sVCAM-1
2009 Kuptniratsaikul [23]	PC, RCT	107	6		2000	↑ Walk distance. ↓ General pain	N/A
2002 Badria [24]	DB, PC, RCT	45	12		500	↑ Walk distance. ↓ General pain	↓ Oxidative stress markers

↑↓ Indicates an increase or decrease in the value of the respective variable, ⇄ Indicates that no change occurred in that respective variable. Abbreviations for Table 1: CGIC: Clinical Global impression of change. CRP: C-reactive protein levels. DB: Double-blind. ESR: Erythrocyte Sedimentation Rate: GI AE: Gastrointestinal adverse event. IL: Interleukin. KOOS: Knee injury and Osteoarthritis Outcome Score. LPFI: Lateral patellar facet impingement. MDA: Malondialdehyde. NSAID: Nonsteroidal anti-inflammatory drug. OBS: Observational study. PC: Placebo controlled. PhyGA: Physician’s global assessment. RCT: Randomized controlled trial. RFT: Renal function tests. ROM: Range of motion. ROS: Reactive oxygen species. SB: Single-blind. sCD40L: Soluble cluster of differentiation 40 ligand. sVCAM: Soluble vascular cell adhesion molecule 1. TUG: Timed up-and-go test. VAS: Visual analogue scale. WOMAC: Western Ontario and McMaster Universities Arthritis Index.

**Table 2:** Clinical efficacy of curcumin supplementation in type 2 diabetes

Study	Study Design	Disease	Trial Size	Study Duration (weeks)	Daily Curcuminoid Intake (mg/day)	Clinical Outcomes in Curcumin Group	Laboratory Outcomes in Curcumin Group
2014 Chuengsamarn [27]	DB, PC, RCT	Diabetes- atherosclerosis	240	24	1500	↓ abdominal obesity (VF + TBF), Atherogenic risks	↑ Serum adiponectin ↓ Pulse wave velocity, leptin, Metabolic profile (triglyceride + uric acid)
2014 Na LX [28]	DB, PC, RCT	Diabetes	100	12	300	N/A	↑ SOD ↓ serum A-FABP, CRP, TNF-a, IL-6
2012 Na LX [29]	DB, PC, RCT	Diabetes	100	12	300	N/A	↑ LPL Activity ↓ Fasting blood glucose, HbA1c, HOMA-IR, serum FFAs + triglycerides
2012 Chuengsamarn [30]	DB, PC, RCT	Diabetes	240	36	1500	↓ T2DM progression	↑ B-cell function (HOMA-B), adiponectin ↓ C-peptide, HOMA-IR, HbA1c, FPG, OGTT at 2 h
2012 Steigerwalt [31]	PC	Diabetes- microangiopathy	38	4	200	↑ Improvement in microangiopathy, visual acuity, ↓ Edema score	↑ Venoarteriolar response
2011 Appendino [32]	PC	Diabetes- microangiopathy	25	4	400	↑ Improvement in microangiopathy, ↓ Skin Flux, ↓ Edema score	↑ PO2, Venoarteriolar response
2011 Khajehdehi [33]	DB, PC, RCT	Diabetes-nephropathy	40	12	66.3	N/A	↓ TGF-B, IL-8, Urinary protein excretion
2008 Usharani [34]	DB, PC, RCT	Diabetes	72	8	300	↑ Endothelial function	↓ MDA, ET-1, IL-6, TNF-a

↑↓ Indicates an increase or decrease in the value of the respective variable. Indicates that no change occurred in that respective variable. A-FABP: Adipokine Adipocyte Fatty Acid-Binding Protein. CRP: C-reactive protein levels. DB: Double-blind. ET-1: Endothelin-1. FFA: Free fatty acids. FPG: Fasting plasma glucose. HBA1C: Hemoglobin A1C. HOMA-B: Homeostatic model assessment for B-cells. HOMA-IR: Homeostatic model assessment for insulin resistance. IL: Interleukin. LPL: Lipoprotein lipase. MDA: Malondialdehyde. OGTT: Oral glucose tolerance test. PC: Placebo controlled. PO2: Partial pressure of oxygen. RCT: Randomized controlled trial. SOD: Superoxide dismutase. TBF: Total body fat. TGF-B: Transforming growth factor beta 1. TNF-A: Tumor necrosis factor alpha. VF: Ventricular fibrillation.

**Table 3:** Clinical efficacy of curcumin supplementation in ulcerative colitis

Study	Study Design	Trial Size	Study Duration (weeks)	Medications	Daily Curcuminoid Intake (mg/day)	Clinical Outcomes in Curcumin Group	Laboratory Outcomes in Curcumin Group
2017 Kedia [36]	DB, PC, RCT	62	8	Mesalamine	450	⇄ Clinical remission, clinical response, mucosal healing, treatment failure	N/A
2015 Lang A [37]	DB, PC, RCT	50	4	Mesalamine	3000	↑ Clinical and endoscopic remission. ↓ SCCAI	N/A
2006 Hanai [38]	DB, PC, RCT, multicenter	89	24	Sulfasalazine or Mesalamine	2000	↓ Clinical activity index, endoscopic index, Relapse occurrence	N/A

↑↓ Indicates an increase or decrease in the value of the respective variable, ⇄ Indicates that no change occurred in that respective variable. Abbreviations: ACR: American College of Rheumatology (criterion). CRP: C-reactive protein levels. DAS: Disease activity score (Part of DAS28). DB: Double-blind. ESR: Erythrocyte sedimentation rate. PC: Placebo controlled. RCT: Randomized controlled trial. SB: Single blind. SCCAI: Simple clinical colitis activity index. SZ: Sulfasalazine. VAS: Visual analogue scale.

**Table 4:** Clinical efficacy of curcumin supplementation in rheumatoid arthritis and lupus nephritis

Study	Study Design	Trial Size	Study Duration (weeks)	Daily Curcuminoid Intake (mg/day)	Clinical Outcomes in Curcumin Group	Laboratory Outcomes in Curcumin Group
2012 Chandran [39]	SB, RCT	45	8	1000	↓ ACR, DAS, VAS	⇄ Blood parameters, ESR. ↓ CRP
1980 Deodhar [40]	DB. RCT	18	2	1300	⇄ DAS	N/A
2012 Khajehdehi [41]	DB, PC, RCT	24	12	66.3	N/A	↓ Proteinuria, systolic blood pressure, hematuria

↑↓ Indicates an increase or decrease in the value of the respective variable, ⇄ Indicates that no change occurred in that respective variable. Abbreviations: ACR: American College of Rheumatology (criterion). CRP: C-reactive protein levels. DAS: Disease activity score (Part of DAS28). DB: Double-blind. ESR: Erythrocyte sedimentation rate. PC: Placebo controlled. RCT: Randomized controlled trial. SB: Single blind. SCCAI: Simple clinical colitis activity index. SZ: Sulfasalazine. VAS: visual analogue scale.
